# Programmed death ligand 1 (PD-L1) in colon cancer and its interaction with budding and tumor-infiltrating lymphocytes (TILs) as tumor-host antagonists

**DOI:** 10.1007/s00384-021-03985-9

**Published:** 2021-06-25

**Authors:** Corinna Lang-Schwarz, Balint Melcher, Arndt Hartmann, Simone Bertz, Theresa Dregelies, Klaus Lang-Schwarz, Michael Vieth, William Sterlacci

**Affiliations:** 1 Institute of Pathology, Klinikum Bayreuth, Preuschwitzer Str. 101, 95445 Bayreuth, Germany; 2 Institute of Pathology, Koblenz, Franz-Weis-Str. 13, 56073 Koblenz, Germany; 3 Institute of Pathology, Friedrich-Alexander-University, Erlangen-Nuremberg, Krankenhausstr. 8-10, 91054 Erlangen, Germany; 4 Department of Anesthesiology, Klinikum Bayreuth, Preuschwitzer Str. 101, 95445 Bayreuth, Germany

**Keywords:** Budding, Tumor-infiltrating lymphocytes (TILs), Colon cancer, PD-L1

## Abstract

**Purpose:**

To analyze the role of programmed death ligand 1 (PD-L1) immunohistochemisty in the context of tumor microenvironment in colon cancer (CC) with focus on the interaction between tumor budding and tumor-infiltrating lymphocytes (TILs) and to elucidate its potential value for immunooncologic treatment decisions.

**Methods:**

Three hundred forty seven patients with CC, stages I to IV, were enrolled. PD-L1 immunohistochemistry was performed using two different antibodies (clone 22C3 pharmDx, Agilent and clone QR1, Quartett). Tumor proportion score (TPS) as well as immune cell score (IC) was assessed. Budding and TILs were assessed according to the criteria of the International Tumor Budding Consensus Conference (ITBCC) and International TILs Working Group (ITWG). Correlation analyses as well as survival analyses were performed.

**Results:**

PD-L1 positivity significantly correlated with TILs > 5% and MMR deficiency, and PD-L1-positive cases (overall and IC) showed significantly longer overall survival (OS) with both antibodies.The parameters “high grade,” “right-sidedness,” and “TILS > 5% regardless of MMR status” evolved as potential parameters for additional immunological treatment decisions. Additionally, TPS positivity correlated with low budding. More PD-L1-positive cases were seen in both high TIL groups. The low budding/high TIL group showed longer disease-free survival and longer OS in PD-L1-positive cases.

**Conclusion:**

Overall, PD-L1 positivity correlated with markers of good prognosis. PD-L1 immunohistochemistry was able to identify parameters as additional potential candidates for immune therapy. Furthermore, it was able to stratify patients within the low budding/high TIL group with significant prognostic impact.

**Supplementary information:**

The online version contains supplementary material available at 10.1007/s00384-021-03985-9.

## Introduction

Colorectal cancer (CRC) is one of the most common cancer types worldwide. In 2020, over 1.9 million people were newly diagnosed with CRC and about 935,000 people died from CRC [1]. Until recently, treatment regimens were based on the tumor node metastasis (TNM) staging system, the grading according to the World Health Organization (WHO) classification and molecular biomarkers [2]. The discovery of immune checkpoint inhibitors has revolutionized cancer treatment regimes, and checkpoint inhibitors have already become part of the therapeutic standard in different human cancer types, for example (but not limited to), lung cancer, malignant melanoma, and breast cancer [3–6].

In mismatch repair-deficient (dMMR) metastatic colon cancer (CC), the progammed death 1 (PD-1) inhibitor pembrolizumab led to a significantly longer progression-free survival than chemotherapy when applied as first-line therapy and showed fewer treatment-related adverse events [7]. Therefore, in June 2020, the US Food and Drug Administration (FDA) approved pembrolizumab (KEYTRUDA, Merck Sharp Dohme) for the first-line treatment of patients with unresectable or metastatic microsatellite instability-high (MSI-H) or dMMR CC, independent from PD-L1 immunohistochemisty [8]. Infact, mismatch repair status seems to be the only reliable feature in CC to predict treatment response to checkpoint inhibitor therapy. So far, studies failed to prove the predictive value of PD-L1 immunohistochemistry in CC.

However, the group of MSI-H or dMMR CC counts for only up to 15% of all patients with CRC, only about 5% of them being stage IV [9–13].

Additionally, in recent years, additive features with focus on tumor microenvironment have gained increasing attention as they have shown potential to predict prognosis or response to therapy or even serve as therapeutic targets.

Among them, on the tumor side, tumor budding, as a morphologic sign of epithelial-mesenchymal transition (EMT), is associated with higher tumor stage (T) and higher nodal status (N), venous invasion (V1) and lymphatic vessel infiltration (L1), local tumor recurrence, distant metastasis, and higher tumor agressiveness [14–25]. It is now accepted as an additional prognostic factor for CRC, according to the Union for International Cancer Control (UICC), and listed among the essential and desirable diagnostic criteria for CRC in the current fifth edition of WHO Classification of Tumours [2, 26].

On the host immunity side, tumor-infiltrating lymphocytes (TILs) are also a popular research object in various cancer types. Increased TILs in CRC are an independent predictor of better prognosis [22, 27, 28]. Assessment and reporting of budding as well as TILs on hematoxylin and eosin (H&E) stained slides have recently been well defined and validated by international groups [29–32]. We could previously show that the combination of tumor budding and TILs as tumor-host antagonists is able to further stratify patients with CC regarding overall survival (OS) and to identify patients in stage II and III CC regarding the benefit from adjuvant chemotherapy [22, 25, 33].

Therefore, the aim of our study was the following:

- To analyze the immunohistochemical PD-L1 staining pattern in a large series of CC, stages I–IV.
- To elucidate the role of PD-L1 immunohistochemistry in the context of the “budding and TIL combination” as tumor-host antagonists.
- To identify PD-L1-positive “budding/TIL” subgroups which might qualify as potential additional candidates for future immunooncogenic treatment decisions.

## Methods

### Case selection

The study cohort consisted of 347 cases of CC, stages I to IV, diagnosed at the Institute of Pathology, Klinikum Bayreuth GmbH, Bayreuth, Germany between 2005 and 2016.

Cases with neoadjuvant treatment modalities and rectal carcinoma (due to high percentage of neoadjuvant treatment) were excluded. Further patient and tumor characteristics are listed in Table 1.

**Table 1:** Summary of patient and tumor characteristics

Feature	Frequency, *n* (%)
Age (year; mean, max, min, *n* = 347)	75 (47–97)
Gender (*n* = 347)	
Male	161 (46.4)
Female	186 (53.6)
pT (*n* = 347)	
pT1	23 (6.6)
pT2	39 (11.2)
pT3	206 (59.4)
pT4	79 (22.8)
pN (*n* = 347) pN0 pN1 pN2	198 (57.1)
	82 (23.6)
	67 (19.3)
M (*n* = 347)	
M0	292 (84.1)
M1	55 (15.9)
TNM stage (*n* = 347) I II III IV	
	51 (14.7) 140 (40.3) 97 (28.0)
	59 (17.0)
Tumor location (right/left, *n* = 347)	
Right	238 (68.6)
Left	109 (31.4)
Grading (WHO 2019, *n* = 347)	
Low grade	277 (79.8)
High grade	70 (20.2)
Venous invasion (*n* = 347)	
V0	274 (79.0)
V1	73 (21.0)
Lymphatic invasion (*n* = 347)	
L0	211 (60.8)
L1	136 (39.2)
Mucinous (y/n; *n* = 347)	
y	24 (6.9)
n (NOS)	323 (93.1)
MMR status (*n* = 312)	
MMR proficient	239 (76.6)
MMR deficient	73 (23.2)
*KRAS* (*n* = 93)	
Wild type	49 (52.7)
Mutated	44 (47.3)

*TNM* tumor node metastasis, *WHO* World Health Organization, *NOS* not otherwise specified, *MMR* mismatch repair, *KRAS* Kirsten rat sarcoma

Follow-up data were provided from the local tumor registry in Bayreuth. A complete follow-up was available for 308 cases. Median follow-up was 30 months (range 0–137 months). One hundred ninety-seven patients were alive at study end; 111 died.

The ethics commission of Friedrich-Alexander-University Erlangen-Nuremberg approved the study (study number 216_19 Bc).

### Histological assessment of budding and TILs

H&E-stained tumor slides of all patients were retrieved from our archives and re-evaluated independently in terms of budding according to the criteria of the ITBCC by two pathologists (CLS, BM) as described previously [25, 33]. Budding was reported as proposed: low budding 0–4 buds (Bd1), intermediate budding 5–9 buds (Bd2), and high budding ≥ 10 buds (Bd3) [29]. Only peritumoral budding at the invasive front was evaluated. For the budding-TIL groups, cases with intermediate (Bd2) and high budding (Bd3) were summarized as one “high budding group” as they had shown a trend to similar overall survival in our previous study [22].

The percentage of tumor-associated lymphatic infiltration was semiquantitatively estimated on the same H&E-stained slides, according to the ITWG methodology and as described before [30, 31]. Referring to our previous studies, the cutoff for the low TILS group was set at ≤ 5% which resulted in four groups [22, 25]:

- Low budding/high TILs (i.e., Bd1 + TILs > 5%).
- Low budding/low TILs (i.e., Bd1 + TILs ≤ 5%).
- High budding/high TILs (i.e., Bd2 or Bd3 and TILs > 5%).
- High budding/low TILs (i.e., Bd2 or Bd3 and TILs ≤ 5%).

### PD-L1 immunohistochemistry

PD-L1 immunohistochemistry was performed on whole tissue sections corresponding to those that had been used for budding and TIL assessment before. Four-micrometer-thick slides were cut, and immunohistochemistry was performed with two different PD-L1 antibodies in order to obtain reproducible results:

- PD-L1 (clone 22C3 pharmDx, monoclonal mouse anti-human, dilution 1:50, Agilent, Santa Clara, CA, USA), which is the routinely used PD-L1 antibody in our laboratory.
- PD-L1 (clone QR1, monoclonal rabbit anti-human, dilution 1:150, Quartett, Berlin, Germany), which the FDA approval for pembrolizumab in dMMR CC is based on.

Immunostaining was performed using the fully automated Bond-III autostainer (Leica Biosystems, Wetzlar, Germany). Antigen retrieval was performed by heat-induced epitope retrieval (HIER) for 20 min at 100 °C with epitope retrieval solution 1 (ER1, citrate buffer, pH 6.0) for PD-L1, clone 22C3 and epitope retrieval solution 2 (ER2, EDTA-based puffer, pH 9.0) for PD-L1 clone QR1. The glass slides were incubated with each antibody for 15 min at room temperature. A Bond Polymer Red Refine Detection System (Leica Microsystems) was used for antibody detection and visualization, using Fast Red as chromogen. All slides were counterstained with hematoxylin for 7 min, dehydrated in ascending grades of alcohol and covered by cover slips with Eukitt. Tonsil tissue served as on-slide positive control.

### PD-L1 assessment

All PD-L1-stained tumor slides were assessed by one pathologist (CLS). The tumor proportion score (TPS) as well as the immune cell score (IC) was measured in percentages from 0 to 100. The TPS is defined as the percentage number of tumor cells with positive membranous staining of any intensity based on all tumor cells. The IC is defined as the percentage number of immune cells (granulocytes, dendritic cells, lymphocytes and macrophages) with positive cytoplasmic staining at any intensity based on all immune cells in the tumor area, including immune cells at the invasive front. Immune cells at the invasive front are included if they are within one field (in 20 × objective magnification) with tumor occupying up to half of the field [34]. Combined positivity score (CPS) was also assessed (results not shown). Prior to the scoring and to learn the scoring method, the pathologist attended several web-based PD-L1 trainings and a password-secured PD-L1 training plattform (Qualitätssicherungs-Initiative Pathologie QuIP GmbH, Berlin, Germany, https://www.pdl1portal.eu/) to assess PD-L1 in line with currently valid scoring conventions.

PD-L1 immunohistochemistry was interpreted as follows: TPS and IC values of ≥ 1% were assigned as positive at any staining itensity. Score values of 0% or < 1% were assigned as negative [34].

**Fig. 1:**
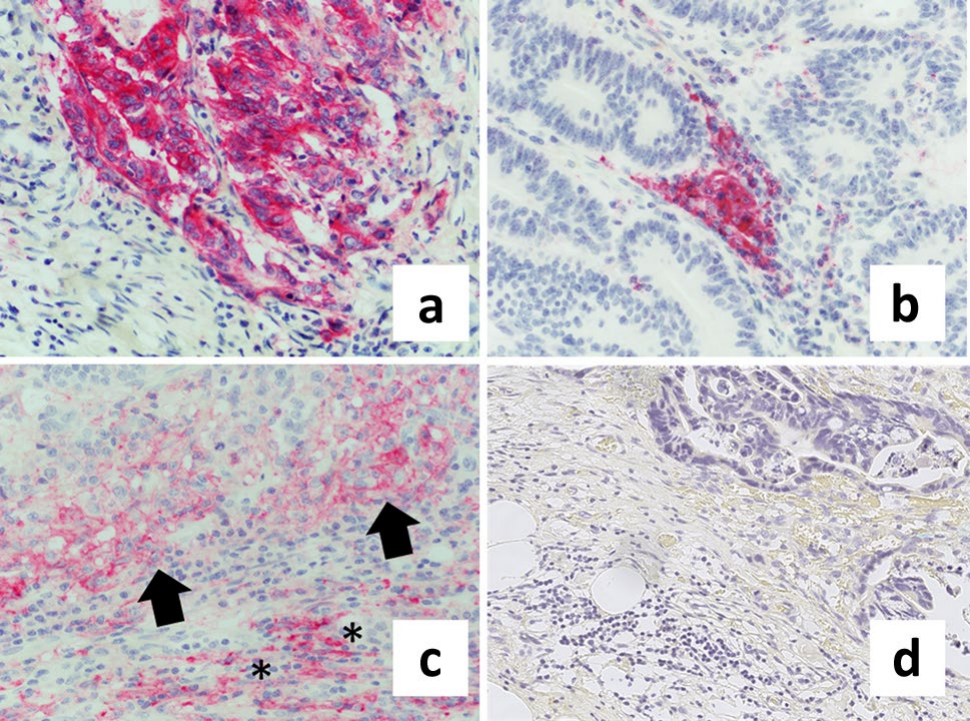
Representative images of PD-L1 immunohistochemistry in colon cancer (CC). **a** TPS positivity, indicated by strong membranous PD-L1 staining of tumor cells in a patient with stage IV, mismatch repair deficient (dMMR) CC with low budding and high TILs (magnification 400 ×). **b** IC positivity, defined as granular cytoplasmic positivity in immune cells in a patient with stage I, dMMR CC with low budding and low TILs (magnification 400 ×). **c** Simultaneous membranous staining of tumor cells (arrows) and granular cytoplasmic staining of immune cells (asterisks) at the tumor invasive front in a patient with stage III, dMMR CC with low budding and high TILs (magnification 400 ×). **d** PD-L1-negative tumor in a patient with stage IV, pMMR CC with high budding and low TILs (magnification 265 ×)

Figure 1 shows histological example images of PD-L1 immunohistochemistry.

For interobserver reliability, a subset of 50, randomly chosen cases, was evaluated by a second pathologist (SW), trained in PD-L1 assessment. Both observers were blinded to each others’ results.

### Statistics

Statistical analyses were performed using the statistics program SPSS 21 (IBM Corp. Released 2012, IBM SPSS Statistics for Windows, Armonk, NY). Pearson’s chi-squared test was used to test the association between different parameters. Interobserver agreement was tested by Cohen’s kappa. Univariate survival analyses for overall survival (OS) were carried out using the Kaplan–Meier method with log-rank test. Multivariate survival analysis was performed using the Cox regression analysis. Hazard ratios and 95% confidence intervals (CIs) were used to determine effect size. *P* values < 0.05 were considered statistically significant.

## Results

### Budding

We found low budding in 266 (76.7%), intermediate budding in 69 (19.9%), and high budding in 12 (3.5%) cases. Higher budding significantly correlated with higher pT (*p* = 0.037) and pN stages (*p* = 0.033), higher TNM stage (*p* = 0.011), nonmucinous CC (not otherwise specified, NOS, *p* = 0.026), L1 (*p* = 0.014), wild-type RAS (*p* = 0.042), and MMR-proficient (pMMR) CC (*p* = 0.040).

Detailed results of the correlation analyses between tumor budding and clinicopathological features are available in Supplemental Table 1.

In Kaplan–Meier analysis, cases with low budding showed significantly longer disease-free survival (DFS) and overall survival (OS) than cases with intermediate budding (*p* = 0.022 and *p* = 0.018). However, no significant difference was found between both groups and high budding.

### TILs

One hundred seventy-seven cases (51%) showed ≤ 5% TILs, and 170 cases (49%) showed > 5% TILs. Higher amounts of TILs were significantly correlated with female gender (*p* = 0.002), less L1 (*p* = 0.001), and less V1 (*p* = 0.001) and correlated highly significant with lower pT (*p* < 0.001), pN (*p* < 0.001), and M stages (*p* < 0.001), as well as nonmucinous (NOS) CC (*p* < 0.001) and lower TNM stages (*p* < 0.001).

Detailed results of the correlation analyses between TILs and clinicopathological features are available in Supplemental Table 2.

In Kaplan–Meier analysis, cases with > 5% TILs showed significantly longer DFS (*p* = 0.015) and OS (*p* = 0.015) compared to cases with ≤ 5% TILs.

### Budding and TILs

One hundred twenty-nine cases belonged to the low budding/high TIL group (37.2%), 128 (36.9%) to the low budding/low TIL group, 42 (12.1%) to the high budding/high TIL group, and 48 cases (13.8%) to the high budding/low TIL group.

The combination of both parameters significantly correlated with gender, pT (*p* < 0.001), pN (*p* < 0.001), M (*p* < 0.001), TNM stage (*p* < 0.001), mucinous versus NOS CC (*p* < 0.001), L1 (*p* < 0.001), V1 (*p* = 0.008), and MMR status (*p* = 0.041). No correlation was found with grading and *KRAS*.

Interobserver agreement between the two pathologists was substantial for budding (*κ* = 0.660, *p* < 0.001) and fair for TILs (*κ* = 0.246, *p* = 0.001).

Detailed results of the correlation analyses between the four budding/TIL groups and clinicopathological features are available as Supplemental Table 3.

In Kaplan–Meier analysis, DFS and OS were best for the low budding/high TIL group (mean DFS 93.439 months, 95% CI 82.122–104.757 months; mean OS 93.862 months, 95% CI 82.703–105.022 months) and worst for the high budding/low TIL group (mean DFS 47.927 months, 95% CI 28.517–67.337 months; mean OS 55.369 months, 95% CI 37.699–73.038 months). Kaplan–Meier analysis of OS for the four budding/TIL groups is shown in the supplemental figure. Differences between the four groups were statistically significant for DFS and OS between the low budding/high TIL group and the low budding/low TIL group (*p* = 0.027 for DFS and *p* = 0.023 for OS) as well as between the low budding/high TIL group and the high budding/low TIL group (*p* = 0.004 for DFS and *p* = 0.005 for OS) and showed a trend to longer OS for low budding/high TILs versus high budding/high TILs (*p* = 0.084).

### PD-L1 immunohistochemistry

Interobserver agreement between the two pathologists was substantial for PD-L1, clone QR (*κ* = 0.623, *p* < 0.001) and fair for PD-L1, clone 22C3 (*κ* = 0.406, *p* = 0.003). Staining was not assessable due to poor slide quality in two cases stained for clone QR and in one case stained for clone 22C3.

PD-L1 staining was completely negative in 182 cases (52.8%) for clone QR and in 241 cases (69.5%) for clone 22C3. Either TPS or IC was positive in 163 cases (47.2%) for clone QR and in 105 cases (30.3%) for clone 22C3. TPS was positive in 61 cases (17.6%) for clone QR and in 25 cases (7.2%) for clone 22C3. IC was positive in 157 cases (45.2%) for clone QR and in 96 cases (27.7%) for clone 22C3. The differences in overall staining positivity, TPS positivity, and IC positivity between both antibodies were statistically significant (*p* < 0.001, *p* < 0.001, *p* < 0.001, respectively).

### PD-L1 IHC, clone QR

With clone QR, positivity in all three settings (overall, TPS, and IC) significantly correlated with TILs > 5% (*p* < 0.001 each), MMR deficiency (*p* < 0.001 each), high grade (*p* = 0.012, *p* = 0.001, and *p* = 0.018), female gender (*p* = 0.025, *p* = 0.027, and *p* = 0.022) and M0 (*p* < 0.001, *p* = 0.047, and *p* < 0.001).

Additionally, overall PD-L1 positivity (TPS and/or IC) with clone QR significantly correlated with lower pN stages (*p* < 0.001), lower TNM stage (*p* < 0.001), right-sided colon cancer (*p* = 0.001), and less V1 (*p* = 0.008). Mucinous tumors were more often PD-L1 overall negative (*p* = 0.050), and cases with L1 showed a trend towards PD-L1 overall negativity (*p* = 0.068).

TPS positivity with clone QR additionally significantly correlated with low budding (*p* = 0.044), nonmucinous tumors (NOS, *p* = 0.008) and showed a trend towards lower pN stages (*p* = 0.072) and right-sided location (*p* = 0.076).

IC positivity with clone QR—in addition to TILs > 5%, dMMR, female gender, high grade, and M0—significantly correlated with pN0 (*p* < 0.001) and lower TNM stage (*p* < 0.001). IC negativity was significantly more frequent in left-sided CC (*p* = 0.001), as well as tumors with L1 (*p* = 0.038) and V1 (*p* = 0.023) and showed a trend towards mucinous tumors (*p* = 0.075).

The results of the correlation analysis with PD-L1 antibody clone QR and the clinicopathological features are shown in Table 2.

**Table 2:** Correlation between PD-L1 antibody, clone QR and the clinicopathological features

	PD-L1 IHC clone QR								
	Overall			TPS			IC		
Feature	Positive (*n*/%)	Negative (*n*/%)	*p* value	Positive (*n*/%)	Negative (*n*/%)	*p* value	Positive (*n*/%)	Negative (*n*/%)	*p* value
Gender Male Female	66 (41.3) 97 (52.4)	94 (58.8) 88 (47.6)	*0.025*	21 (13.0) 40 (21.5)	140 (87.0) 146 (78.5)	*0.027*	63 (39.1) 94 (50.5)	98 (60.9) 92 (49.5)	*0.022*
pT 1 2 3 4	9 (40.9) 20 (41.3) 104 (50.7) 30 (38.0)	13 (59.1) 19 (48.7) 101 (49.3) 49 (62.0)	0.228	3 (13.0) 5 (12.8) 40 (19.4) 13 (16.5)	20 (87.0) 34 (87.2) 166 (80.6) 66 (83.5)	0.686	9 (39.1) 20 (51.3) 99 (48.1) 29 (36.7)	14 (60.9) 19 (48.7) 107 (51.9) 50 (63.3)	0.272
pN 0 1 2	108 (54.5) 42 (47.5) 17 (25.4)	90 (45.5) 38 (52.5) 50 (74.6)	< *0.001*	36 (18.2) 19 (23.2) 6 (9.0)	162 (81.8) 63 (76.8) 61 (91.0)	0.072	105 (53.0) 36 (43.9) 16 (23.9)	93 (47.0) 46 (56.1) 51 (76.1)	< *0.001*
M 0 1	154 (53.1) 9 (16.4)	136 (46.9) 46 (83.6)	< *0.001*	56 (19.2) 5 (9.1)	236 (80.8) 50 (90.9)	*0.047*	149 (51.0) 8 (14.5)	143 (49.0) 47 (85.5)	< *0.001*
Grading Low grade High grade	121 (44.0) 42 (60.0)	154 (56.0) 28 (40.0)	*0.012*	39 (14.1) 22 (31.4)	238 (95.9) 48 (68.6)	*0.001*	117 (42.2) 40 (57.1)	160 (57.8) 30 (42.9)	*0.018*
Mucinous Yes No (NOS)	7 (29.2) 156 (58.6)	17 (70.8) 165 (51.4)	0.050	0 (0.0) 61 (18.9)	24 (100.0) 262 (81.1)	*0.008*	7 (29.2) 150 (46.4)	17 (70.8) 173 (53.6)	0.075
TNM stage I II III IV	25 (49.0) 83 (59.3) 45 (47.4) 10 (16.9)	26 (51.0) 57 (40.7) 50 (52.6) 49 (83.1)	< *0.001*	7 (13.7) 30 (21.4) 19 (19.6) 5 (8.5)	44 (86.3) 110 (78.6) 78 (80.4) 54 (91.5)	0.133	25 (49.0) 80 (57.1) 43 (44.3) 9 (15.3)	26 (51.0) 60 (42.9) 54 (55.7) 50 (84.7)	< *0.001*
Localization Right-sided Left-sided	126 (53.4) 37 (33.9)	110 (46.6) 72 (66.1)	*0.001*	47 (19.7) 14 (12.8)	191 (80.3) 95 (87.2)	0.076	121 (50.8) 36 (33.0)	117 (49.2) 73 (67.0)	*0.001*
L 0 1	106 (50.7) 57 (41.99	103 (41.9) 79 (58.1)	0.068	35 (16.6) 26 (19.1)	176 (83.4) 110 (80.9)	0.321	104 (49.3) 53 (39.0)	107 (50.7) 83 (61.0)	*0.038*
V 0 1	138 (50.7) 25 (34.2)	134 (49.3) 48 (65.8)	*0.008*	49 (17.9) 12 (16.4)	225 (82.1) 61 (83.6)	0.463	132 (48.2) 25 (34.2)	142 (51.8) 48 (65.8)	*0.023*
*KRAS* Wild type Mutated	19 (39.6) 16 (36.4)	29 (60.4) 28 (63.6)	0.459	5 (10.2) 4 (9.1)	44 (89.8) 40 (90.9)	0.569	19 (38.8) 15 (34.1)	30 (61.2) 29 (65.9)	0.401
MMR Proficient Deficient	101 (42.4) 48 (65.8)	137 (57.6) 25 (34.2)	< *0.001*	31 (13.0) 23 (31.5)	208 (87.0) 50 (68.5)	< *0.001*	97 (40.6) 47 (64.4)	142 (59.4) 26 (35.6)	< *0.001*
Budding Low Intermediate High	130 (49.2) 30 (43.5) 3 (25.0)	134 (50.8) 39 (56.5) 9 (75.0)	0.098	52 (19.5) 9 (87.0) 0 (0.0)	214 (80.5) 60 (13.0) 12 (100.0)	*0.044*	126 (47.4) 28 (40.6) 3 (25.0)	140 (52.6) 41 (59.4) 9 (75.0)	0.091
TILs ≤ 5% > 5%	63 (35.8) 100 (59.2)	113 (64.2) 69 (40.8)	< *0.001*	15 (8.5) 46 (27.1)	162 (91.5) 124 (72.9)	< *0.001*	60 (33.9) 97 (57.1)	117 (66.1) 73 (42.9)	< *0.001*

Statistically significant values are indicated in italics

*IHC* immunohistochemistry, *TPS* tumor positivity score, *IC* immunocell score, *TILs* tumor-infiltrating lymphocytes, *TNM* tumor node metastasis, *KRAS* Kirsten rat sarkoma, *MMR* mismatch repair

### PD-L1 IHC, clone 22C3

Concerning PD-L1 antibody clone 22C3, TILs > 5% and MMR deficiency were the only two clinicopathological features that were significantly associated with PD-L1 positivity in all three measures (overall, TPS, and IC; *p* values < 0.001 each for TILs > 5% and MMR deficiency). Left-sidedness of CC was the only feature that was associated with PD-L1-negative tumors in all three measures (*p* = 0.027, *p* = 0.005, *p* = 0.039, respectively).

Apart from left-sidedness, overall 22C3 negativity was significantly associated with higher pT stages (*p* = 0.001), higher pN stages (*p* = 0.016), higher TNM stages (*p* < 0.001), and V1 (*p* = 0.002). TPS was significantly more often negative in male patients (*p* = 0.044) and low-grade tumors (*p* < 0.001).

IC negativity correlated with higher pT (*p* < 0.001) and pN stages (*p* = 0.013), higher TNM stage (*p* < 0.001), V1 (*p* = 0.009), and higher budding (*p* = 0.022).

The results of the correlation analysis with PD-L1 antibody clone, 22C3 and the clinicopathological features are shown in Table 3.

**Table 3:** Correlation between PD-L1 antibody, clone 22C3 and the clinicopathological features

	PD-L1 IHC, clone 22C3								
	Overall			TPS			IC		
Feature	Positive (*n*/%)	Negative (*n*/%)	*p* value	Positive (*n*/%)	Negative (*n*/%)	*p* value	Positive (*n*/%)	Negative (*n*/%)	*p* value
Gender Male Female	50 (31.3) 55 (29.6)	110 (68.8) 131 (70.4)	0.412	7 (4.4) 18 (9.7)	153 (95.6) 168 (90.3)	*0.044*	47 (29.4) 49 (26.3)	113 (70.6) 137 (73.7)	0.306
pT 1 2 3 4	2 (8.7) 14 (35.9) 76 (36.9) 13 (16.7)	21 (91.3) 25 (64.1) 130 (63.1) 65 (83.3)	*0.001*	1 (4.3) 1 (2.6) 16 (7.8) 7 (9.0)	22 (95.7) 38 (97.4) 190 (92.2) 71 (91.0)	0.574	2 (8.7) 14 (35.9) 71 (34.5) 9 (11.5)	21 (91.3) 25 (64.1) 135 (65.5) 69 (88.5)	< *0.001*
pN 0 1 2	72 (36.4) 20 (24.4) 13 (19.7)	126 (63.6) 62 (75.6) 53 (80.3)	*0.016*	16 (8.1) 5 (6.1) 4 (6.1)	182 (91.9) 77 (93.9) 62 (93.9)	0.777	67 (33.8) 17 (20.7) 12 (18.2)	131 (66.2) 65 (79.3) 54 (81.8)	*0.013*
M 0 1	101 (34.6) 4 (7.4)	191 (65.4) 50 (92.6)	< *0.001*	23 (7.9) 2 (3.7)	269 (92.1) 52 (96.3)	0.218	93 (31.8) 3 (5.6)	199 (68.2) 51 (94.4)	< *0.001*
Grading Low grade High grade	81 (29.3) 24 (34.3)	195 (70.7) 46 (65.7)	0.254	11 (4.0) 14 (20.0)	265 (96.0) 56 (80.0)	< *0.001*	78 (28.3) 18 (25.7)	198 (71.7) 52 (74.3)	0.396
Mucinous Yes No (NOS)	6 (25.0) 99 (30.7)	18 (75.0) 223 (69.3)	0.368	0 (0.0) 25 (7.8)	24 (100.0) 297 (92.2)	0.155	6 (25.0) 90 (28.0)	18 (75.0) 232 (72.0)	0.482
TNM stage I II III IV	14 (27.5) 58 (41.4) 28 (29.9) 5 (8.6)	37 (72.5) 82 (58.6) 69 (91.1) 53 (91.4)	< *0.001*	2 (3.9) 14 (10.0) 7 (7.2) 2 (3.4)	49 (96.1) 126 (90.0) 90 (92.8) 56 (96.6)	0.299	14 (27.5) 53 (37.9) 25 (25.8) 4 (6.9)	37 (72.5) 87 (62.1) 72 (74.2) 54 (93.1)	< *0.001*
Localization Right-sided Left-sided	80 (33.8) 25 (22.9)	157 (66.2) 84 (77.1)	*0.027*	23 (9.7) 2 (1.8)	214 (90.3) 107 (98.2)	*0.005*	73 (30.8) 23 (23.1)	164 (69.2) 86 (78.90)	*0.039*
L 0 1	70 (33.2) 35 (25.9)	141 (66.8) 100 (74.1)	0.094	14 (6.6) 11 (8.1)	197 (93.4) 124 (91.9)	0.371	65 (30.8) 31 (23.0)	146 (69.2) 104 (77.0)	0.071
V 0 1	93 (34.1) 12 (16.4)	180 (65.9) 61 (83.6)	*0.002*	21 (7.7) 4 (5.5)	252 (92.3) 69 (94.5)	0.361	84 (30.8) 12 (16.4)	189 (69.2) 61 (83.6)	*0.009*
*KRAS* Wild type Mutated	10 (20.4) 8 (18.6)	39 (79.6) 35 (81.4)	0.520	4 (8.2) 2 (4.7)	45 (91.8) 41 (95.3)	0.403	7 (14.3) 8 (18.6)	42 (85.7) 35 (81.4)	0.390
MMR Proficient Deficient	63 (26.4) 35 (47.9)	176 (73.6) 38 (52.1)	< *0.001*	9 (3.8) 14 (19.2)	230 (96.2) 59 (80.8)	< *0.001*	97 (40.6) 47 (64.4)	142 (59.4) 26 (35.6)	< *0.001*
Budding Low Intermediate High	130 (49.2) 30 (43.5) 3 (25.0)	134 (50.8) 39 (56.5) 9 (75.0)	0.098	21 (7.9) 3 (4.3) 1 (8.3)	244 (92.1) 66 (95.7) 11 (91.7)	0.490	82 (30.0) 12 (17.4) 2 (16.7)	183 (69.1) 57 (82.6) 10 (83.3)	*0.022*
TILs ≤ 5% > 5%	63 (35.8) 100 (59.2)	113 (64.2) 69 (40.8)	< *0.001*	15 (8.5) 46 (27.1)	162 (91.5) 124 (92.9)	< *0.001*	60 (33.9) 97 (57.1)	117 (66.1) 73 (42.9)	< *0.001*

Statistically significant values are indicated in italics

*IHC* immunohistochemistry, *TPS* tumor positivity score, *IC* immune cell score, *TILs* tumor-infiltrating lymphocytes, *TNM* tumor node metastasis, *KRAS* Kirsten rat sarkoma, *MMR* mismatch repair

In Kaplan–Meier survival analysis, cases with PD-L1 IC positivity showed significantly longer OS compared to PD-L1-negative cases with both antibodies (*p* = 0.006 for clone QR and *p* = 0.002 for clone 22C3). The benefit in OS was even higher in the overall positivity cases (IC and/or TPS, *p* = 0.001 for each antibody).

The results of the analysis between both antibodies and the four budding/TIL groups are shown in Table 4 (clone QR) and Table 5 (clone 22C3). With both antibodies, PD-L1-positive cases in both high TIL groups were significantly more frequent in the three PD-L1 categories (overall, IC, TPS) compared to both low TIL groups (*p* < 0.001 each for clone QR and *p* < 0.001, *p* = 0.002 and *p* < 0.001 for clone 22C3, respectively).

**Table 4:** Correlation between PD-L1-antibody, clone QR and the four budding/TIL groups

	PD-L1 IHC, clone QR								
	Overall			TPS			IC		
Budding/TIL groups	Positive (*n*/%)	Negative (*n*/%)	*p* value	Positive (*n*/%)	Negative (*n*/%)	*p* value	Positive (*n*/%)	Negative (*n*/%)	*p* value
Low buds/high TILs (*n* = 129)	76 (59.4)	52 (40.6)	< *0.001*	39 (30.2)	90 (69.8)	< *0.001*	74 (57.4)	55 (42.6)	< *0.001*
Low buds/low TILs (*n* = 128)	50 (39.4)	77 (60.6)		10 (7.8)	118 (92.2)		48 (37.5)	80 (62.5)	
High buds/high TILs (*n* = 42)	24 (57.1)	18 (42.9)		7 (16.7)	35 (83.3)		23 (54.8)	19 (45.2)	
High buds/low TILs (*n* = 48)	13 (27.1)	35 (72.9)		5 (10.4)	43 (89.6)		12 (25.0)	36 (75.0)	

Statistically significant values are indicated in italics

*IHC* immunohistochemisty, *TPS* tumor positivity score, *IC* immune cell score, *TILs* tumor-infiltrating lymphocytes

**Table 5:** Correlation between PD-L1-antibody, clone 22C3 and the four budding/TIL groups

	PD-L1 IHC, clone 22C3								
	Overall			TPS			IC		
Budding/TIL groups	Positive (*n*/%)	Negative (*n*/%)	*p* value	Positive (*n*/%)	Negative (*n*/%)	*p* value	Positive (*n*/%)	Negative (*n*/%)	*p* value
Low buds/high TILs (*n* = 129)	60 (46.5)	69 (43.5)	< *0.001*	18 (14.0)	111 (86.0)	*0.002*	54 (41.9)	75 (58.1)	< *0.001*
Low buds/low TILs (*n* = 128)	28 (22.0)	99 (78.0)		3 (2.4)	124 (97.6)		26 (20.5)	101 (79.5)	
High buds/high TILs (*n* = 42)	11 (26.2)	31 (73.8)		3 (7.1)	39 (92.9)		10 (23.8)	32 (76.2)	
High buds/low TILs (*n* = 48)	6 (12.5)	42 (87.5)		1 (2.1)	47 (97.9)		6 (12.5)	42 (87.5)	

Statistically significant values are indicated in italics

*IHC* immunohistochemistry, *TPS* tumor positivity score, *IC* immune cell score, *TILs* tumor-infiltrating lymphocytes

**Fig. 2:**
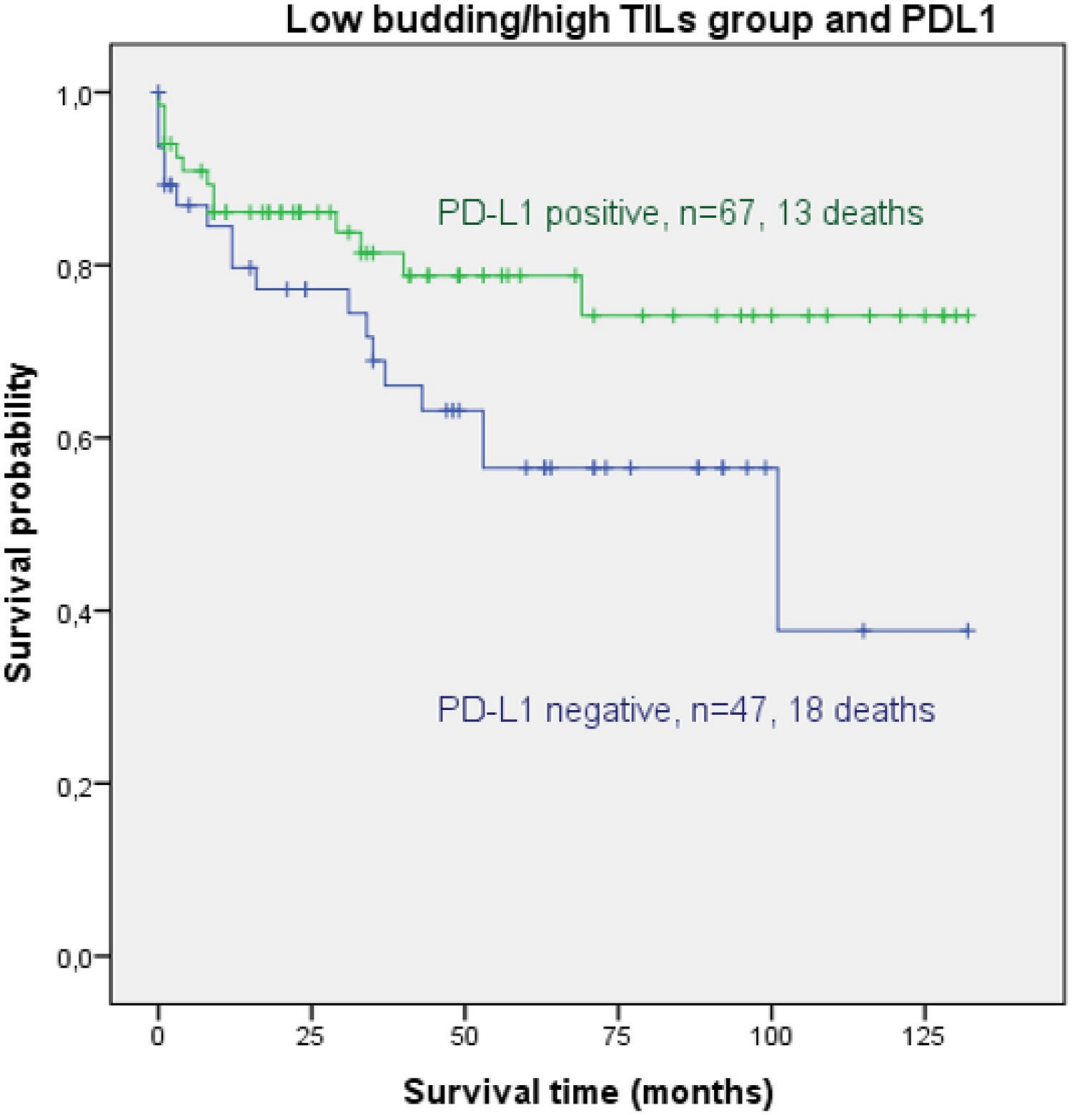
Kaplan–Meier analysis showing overall survival (OS) for the low budding/high TIL group dependent on PD-L1 overall positivity (TPS and/or IC positive) with clone QR. The difference between the PD-L1-positive cases (blue) and the PD-L1-negative cases (green) was statistically significant (*p* = 0.049)

Concerning Kaplan–Meier analysis, with clone QR, the low bud/high TIL group was the only group that showed a significantly longer DFS and OS in the case of overall PD-L1 positivity compared to PD-L1-negative cases (*p* = 0.045 for DFS and *p* = 0.049 for OS), whereas no differences were seen for TPS and IC as well as the other three budding/TIL groups. Kaplan–Meier analysis for OS for the low budding/high TIL group is shown as Fig. 2.

**Fig. 3:**
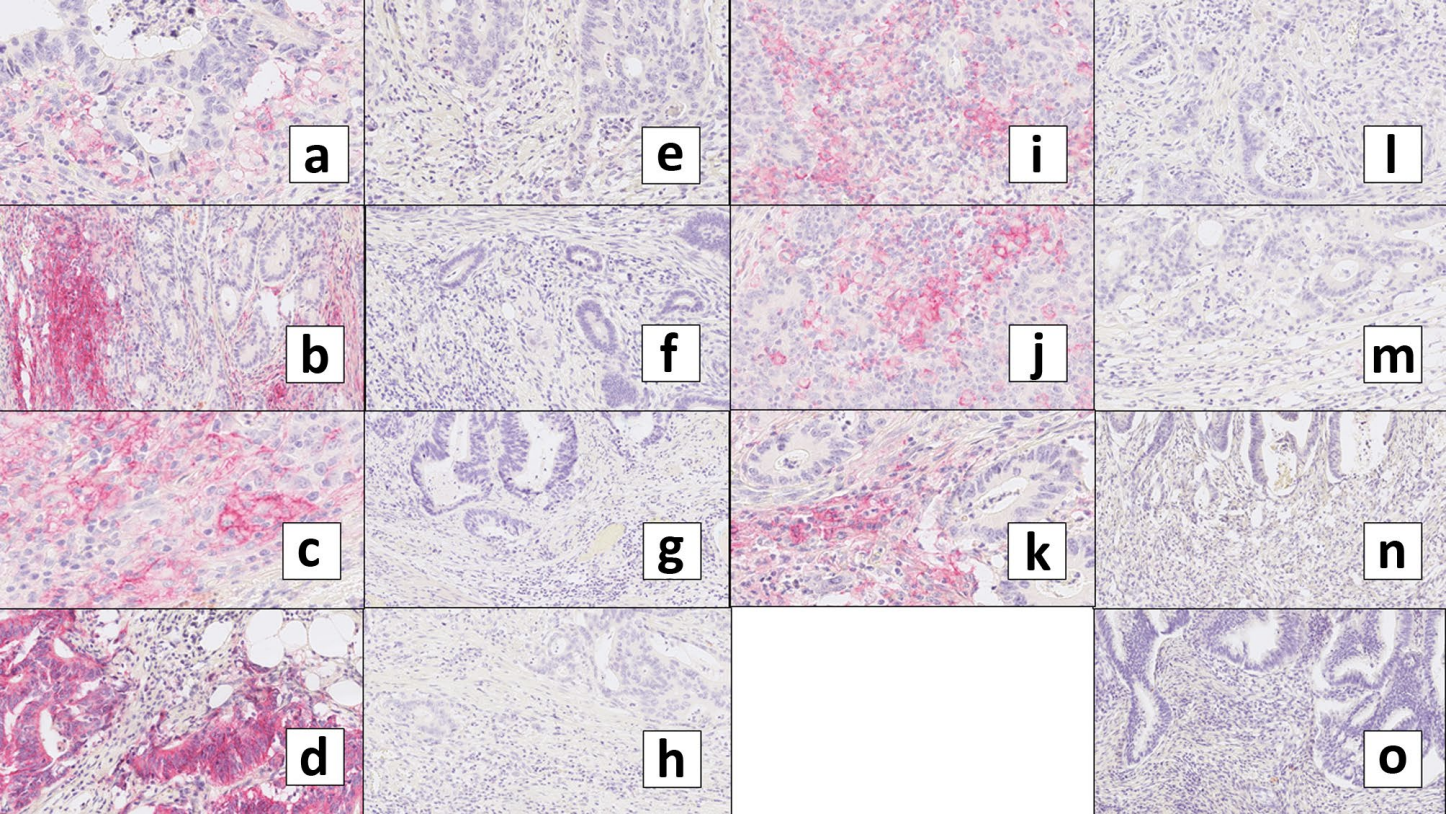
Representative histological images of PD-L1 immunohistochemistry (overall positive versus negative) for the low budding/high TIL group for each tumor stage (I–IV) and further separated into right-sided (**a–h**) versus left-sided (**i–o**). As only 8 out of 129 patients in the low budding/high TIL group belonged to stage IV CC, there was no case with PD-L1 positivity in the left-sided hemicolon in stage IV. **a–d** PD-L1 positive, right-sided (**a** stage I, **b** stage II, **c** stage III, **d** stage IV). **e–h** PD-L1 negative, right-sided (**e** stage I, **f** stage II, **g** stage III, **h** stage IV). **i–k** PD-L1 positive, left-sided (**i** stage I, **j** stage II, **k** stage III). **l–o** PD-L1 negative, left-sided (**l** stage I, **m** stage II, **n** stage III, **o** stage IV)

Figure 3 shows representative histological images of PD-L1 immunohistochemistry (overall positive versus negative) for the low budding/high TIL group for each tumor stage (I–IV) and further separated into right-sided versus left-sided. As only 8 out of 129 patients in the low budding/high TIL group had stage IV CC, there was no case with PD-L1 positivity in the left-sided hemicolon.

With clone 22C3, a trend was seen towards better DFS and OS in low budding/high TIL cases with IC positivity (*p* = 0.053 for DFS and *p* = 0.067 for OS). However, no significant differences were seen in overall positivity, TPS, and the other three budding/TIL groups.

### MMR-proficient cases

MMR status was available for 312 cases. Among these, 239 (76.6) were pMMR and 73 (23.4%) were dMMR.

The results of the correlation analysis between the four budding/TIL groups in pMMR cases and both PD-L1 antibodies are shown in Table 6 (clone QR) and Table 7 (clone 22C3). With both antibodies, cases in both high-TIL groups significantly correlated with PD-L1 overall and IC positivity (*p* = 0.008 and *p* = 0.003 for clone QR and *p* = 0.002 each for clone 22C3). Both high-TIL groups showed longer OS in Kaplan–Meier analysis in PDL1-positive cases. However, the differences in OS between PD-L1 positive and negative cases in each of the four budding/TIL groups were not statistically significant.

**Table 6:** Correlation between the four budding/TIL groups in pMMR cases and PD-L1 antibody, clone QR

	PD-L1 IHC, clone QR, MMR-proficient cases								
	Overall			TPS			IC		
Budding/TIL groups	Positive (*n*/%)	Negative (*n*/%)	*p* value	Positive (*n*/%)	Negative (*n*/%)	*p* value	Positive (*n*/%)	Negative (*n*/%)	*p* value
Low buds/high TILs (*n* = 129)	44 (53.0)	39 (47.0)	*0.008*	16 (19.3)	67 (80.7)	0.181	44 (53.0)	39 (47.0)	*0.003*
Low buds/low TILs (*n* = 128)	29 (34.1)	56 (65.9)		7 (8.1)	79 (91.9)		27 (31.4)	59 (68.6)	
High buds/high TILs (*n* = 42)	18 (54.5)	15 (45.5)		4 (12.1)	29 (87.9)		17 (51.5)	16 (48.5)	
High buds/low TILs (*n* = 48)	10 (27.0)	27 (73.0)		4 (10.8)	33 (89.2)		9 (24.3)	28 (75.7)	

Statistically significant values are indicated in italics

*IHC* immunohistochemistry, *TPS* tumor positivity score, *IC* immunecell score, *TILs* tumor-infiltrating lymphocytes

**Table 7:** Correlation between the four budding/TIL groups in pMMR cases and PD-L1 antibody, clone 22C3

	PD-L1 IHC, clone 22C3, MMR-proficient cases								
	Overall			TPS			IC		
Budding/TIL groups	Positive (*n*/%)	Negative (*n*/%)	*p* value	Positive (*n*/%)	Negative (*n*/%)	*p* value	Positive (*n*/%)	Negative (*n*/%)	*p* value
Low buds/high TILs (*n* = 129)	34 (41.0)	49 (59.0)	*0.002*	4 (4.8)	79 (95.2)	0.719	32 (38.6)	51 (61.4)	*0.002*
Low buds/low TILs (*n* = 128)	16 (18.6)	70 (81.4)		2 (2.3)	84 (97.7)		14 (16.3)	72 (83.7)	
High buds/high TILs (*n* = 42)	8 (24.2)	25 (75.8)		2 (6.1)	31 (93.9)		7 (21.2)	26 (78.8)	
High buds/low TILs (*n* = 48)	5 (13.5)	32 (86.5)		1 (2.7)	36 (97.3)		5 (13.5)	32 (86.5)	

Statistically significant values are indicated in italics

*IHC* immunohistochemistry, *TPS* tumor positivity score, *IC* immunecell score, *TILs* tumor-infiltrating lymphocytes

Multivariate analysis revealed independent prognostic effects of PD-L1 positivity regarding pT (*p* < 0.001), pN (*p* < 0.001), M (*p* < 0.001), TNM stage (*p* < 0.001), grading (*p* = 0.026), and V1 (*p* < 0.001) and showed a trend towards TILs (*p* = 0.076) and the budding/TIL combination (*p* = 0.084, Table 8).

**Table 8:** Results of the multivariate analysis for clinical features, budding, TILs, and PD-L1 immunohistochemistry

		PD-L1 positivity	
Feature		HR (95% CI)	*p* value
pT	4 1 2 3	1.0 0.510 (0.239–1.102) 0.148 (0.046–0.480) 0.481 (0.320–0.722)	< *0.001*
pN	2 0 1	1.0 0.400 (0.256–0.626) 0.457 (0.272–0.769)	< *0.001*
M	1 0	1.0 0.256 (0.166–0.395)	< *0.001*
TNM stage	IV I II III	1.0 0.203 (0.097–0.427) 0.229 (0.137–0.382) 0.285 (0.173–0.470)	< *0.001*
Grading (WHO)	High grade Low grade	1.0 0.586 (0.374–0.920)	*0.026*
V	1 0	1.0 0.448 (0.300–0.667)	< *0.001*
L	1 0	1.0 0.744 (0.511–1.085)	0.126
MMR status	dMMR pMMR	1.0 1.472 (0.834–2.597)	0.166
Budding	high low intermediate	1.0 1.051 (0.331–3.339) 1.688 (0.514–5.545)	0.111
TILs	> 5%	1.0	0.076
	≤ 5%	1.426 (0.960–2.118)	
Score budding/TILs	High buds/low TILs Low buds/high TILs Low buds/low TILs High buds/high TILs	1.0 0.545 (0.310–0.960) 0.863 (0.516–1.444) 1.016 (0.504–2.050)	0.084

Statistically significant values are indicated in italics

*HR* hazard ratio, *CI* confidence intervall, *TILs* tumor-infiltrating lymphocytes, *TPS* tumor positivity score, *IC* immune cell score, *TNM* tumor node metastasis, *MMR* mismatch repair

## Discussion

PD-L1 immunohistochemistry is mandatory for the decision proimmunooncogenic or contraimmunooncogenic treatment in tumors of different organs, for example, bladder cancer, lung cancer, or breast cancer [4, 35–38]. For CC, in June 2020, the FDA approved pembrolizumab (KEYTRUDA, Merck Sharp Dohme) for first-line treatment of patients with unresectable or metastatic MSI-H or dMMR CRC, independent of PD-L1 immunohistochemisty [8].

However, the majority of CC patients belong to stages I to III and/or pMMR (in this cohort: dMMR in 23.4%, only 1.9% thereof in stage IV versus 76.6% pMMR, stages I to IV). Therefore, there is a need to identify additional patients with CC who might benefit from immune therapy on the basis of their tumor biology and features of tumor microenvironment might be helpful for this issue.

The idea of analyzing budding (on the tumor side) and TILs (on the host side) as attacker-defender approach had was first described by Lugli et al. in 2009 [39].

In our previous studies, we were able to show that the combination of tumor budding and TILs as tumor-host antagonists is able to stratifiy patients with CC into prognostic subgroups with different OS. The parameter TILs proved to be more relevant regarding prognosis than the parameter budding. However, budding was also able to further stratify the low TIL cases into subgroups with different OS [22, 25]. Recently, we could further show that the budding/TIL combination is able to identify patients in stage II and III CC with and without benefit from adjuvant treatment [33].

Therefore, we aimed to analyze the interaction of PD-L1 immunohistochemistry with the budding/TIL combination to determine its potential value for the identification of additional candidates for immune therapy.

Studies focussing on PD-L1 immunohistochemistry in CC are very heterogenous in their scoring conventions, regarding, for example, amount of tissue for PD-L1 assessment (tissue micro array versus whole tumor slide), staining pattern (membranous versus cytoplasmatic), and cutoffs for PD-L1 positivity which make them difficult to compare. Elfishawy et al., for example, measured membranous staining only in tumor cells and stromal TILs and defined positivity as > 5% [40]. Wang et al. assessed PD-L1 in duplicate cores of 1 mm each on tissue micro arrays and also only evaluated membranous staining on tumor cells as well as immune cells, using stepwise cutoffs from < 1 to > 10% [41].

Moreover, differences in staining quantities between different antibodies and staining platforms are well known, and harmonization trials are conducted in order to provide recommendations for their use and different interpretations [42, 43]. When comparing the herein used two antibodies, with clone QR, more positive cases were found in PD-L1 overall positivity, TPS, and IC (47.2%, 17.6%, and 45.2%, respectively) compared to clone 22C3 (30.3%, 7.2%, and 27.7%, respectively). In our study, TILs > 5% and dMMR were the only two parameters that correlated reproducible and significantly with PD-L1 positivity in all three settings (overall, TPS, IC) with both antibodies.

Interestingly, overall and IC PD-L1 positivity with both antibodies were significantly linked to more favourable clinicopathological features like lower pT, pN, and M and less L1 and V1, as well as lower tumor stage. PD-L1-positive cases (overall and IC) showed better OS. Wyss et al. found similar results in their study on 279 patients with CC. Stromal PD-L1 and PD-1 expressions were both associated with less aggressive tumor behavior and better OS and DFS [44]. Some studies showed a correlation between lower T stages, pN0, lower T stage, and PD-L1 positivity too; however, others did not [45–47]. Interestingly, PD-L1 positivity was also correlated with high grade and right-sidedness of CC which has also been shown by Wang et al. Kim et al. Elfishawy et al. and Lee et al. [40, 41, 48, 49].

Regarding the four budding/TIL groups, not surprisingly, PD-L1 positivity was statistically significantly more frequent in both high TIL groups. However, this was also the case when dMMR cases were excluded, allowing for the hypothesis that tumors with high TILs might respond to immune therapy even in the case of pMMR.

Interestingly, in our cohort, TILs did not correlate to MMR status but were almost equally distributed on dMMR and pMMR cases, which can probably be explained by our low cutoff for TIL stratification of 5% and which is also in line with the study of Fuchs et al. on their large cohort of more than 1000 CC patients, who also used the ITWG method for TIL assessment [32].

Most interestingly, the low budding/high TIL group was the only group that showed significantly better DFS and OS in the cases of PD-L1 overall positivity with clone QR (and a trend towards better DFS and OS with 22C3). This is interesting, as PD-L1 immunohistochemistry was able to further subdivide this group, which has already been shown to have the best OS of all four budding/TIL groups in our previous studies and is one of the two groups representing most CC cases [22].

## Conclusion

As the outcome of patients with CC differs even in tumors with identical TNM stage, the focus of interest has switched to further characterization of tumor microenvironment in recent years.

While elucidating the role of PD-L1 immunohistochemistry in CC with focus on its interaction with the budding and TIL combination, we were able to identify high grade, right-sidedness of CC, and tumors with TILs > 5%, regardless of MMR status as parameters that might have potential to identify additional candidates that might benefit from immune therapy in CC. Additionally, patients with PD-L1 positivity in the low budding/high TIL group showed significantly better OS than PD-L1-negative cases. Further studies must show if patients in this group really have benefit from immune therapy. To the best of our knowledge, this is the first study analyzing the role of PD-L1 immunohistochemistry in the context of tumor budding and TILs as tumor-host antagonists. Further studies are necessary to elucidate if parameters of the tumor microenvironment can help in identifying patients with potential benefit from immune therapy in CRC, as there is currently no such option for the majority of CRC patients.

## Supplementary information

Below is the link to the electronic supplementary material.

Supplementary file1 (TIF 241 KB)

Supplementary file2 (DOCX 15 KB)

Supplementary file3 (DOCX 16 KB)

Supplementary file4 (DOCX 15 KB)

## Data Availability

Data, as far as not anyway shown, are available on demand from the corresponding author via e-mail: Corinna.Lang-Schwarz@klinikum-bayreuth.de.
