# Supplementary figures and images for: Programmed death ligand 1 (PD-L1) in colon cancer and its interaction with budding and tumor-infiltrating lymphocytes (TILs) as tumor-host antagonists
Source: Int J Colorectal Dis. 2021 Jun 25;36(11):2497–510. doi: 10.1007/s00384-021-03985-9 (PMC8505298; doi:10.1007/s00384-021-03985-9)

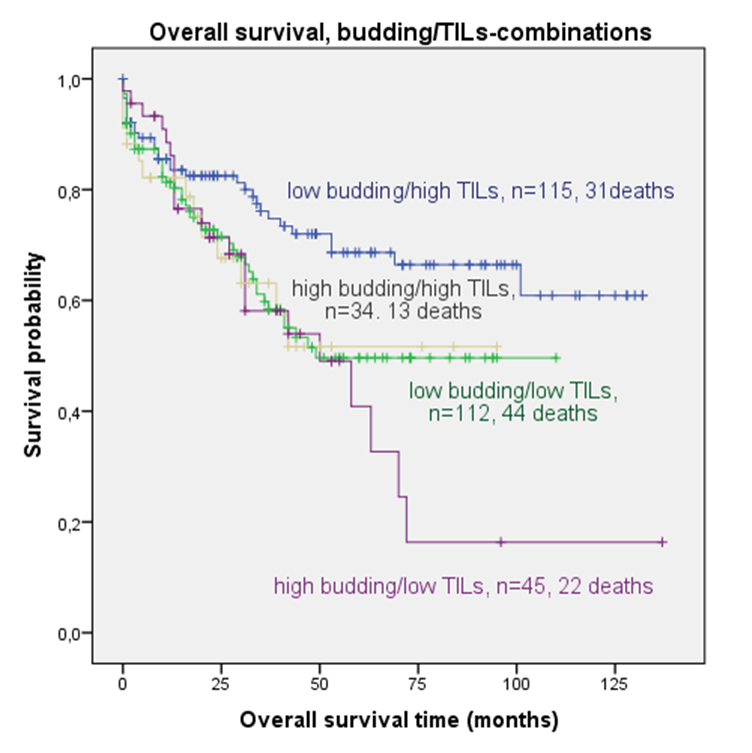

Supplement: Supplementary file 1 — Supplementary file1 (TIF 241 KB) [file 384_2021_3985_MOESM1_ESM.tif]
