# Supplementary material for: Programmed death ligand 1 (PD-L1) in colon cancer and its interaction with budding and tumor-infiltrating lymphocytes (TILs) as tumor-host antagonists
Source: Int J Colorectal Dis. 2021 Jun 25;36(11):2497–510. doi: 10.1007/s00384-021-03985-9 (PMC8505298; doi:10.1007/s00384-021-03985-9)
Supplement: Supplementary file 2 — Supplementary file2 (DOCX 15 KB) [file 384_2021_3985_MOESM2_ESM.docx]

| **Feature** |  | **Low budding (n/%)** | | **Intermediate budding (n/%)** | | **High budding (n/%)** | **p-value** |
| --- | --- | --- | --- | --- | --- | --- | --- |
|  |  | 75 | | 76 | 70 | |  |
| Age (yrs) | mean |  |  |  |  |  | 0.862 |
| Gender |  |  |  |  |  |  |  |
|  | male | 122 (75.8) | | 32 (19.9) | | 7 (4.3) | 0.553 |
|  | female | 144 (77.4) | | 37 (19.9) | | 5 (2.7) |  |
| pT |  |  |  |  |  |  |  |
|  | 1 | 22 (95.7) | | 1 (4.3) | | 0 (0.0) | *0.037* |
|  | 2 | 32 (82.1) | | 4 (10.3) | | 3 (7.7) |  |
|  | 3 | 155 (75.2) | | 46 (22.3) | | 5 (2.4) |  |
|  | 4 | 57 (72.2) | | 18 (22.8) | | 4 (5.1) |  |
| pN |  |  |  |  |  |  |  |
|  | 0 | 160 (80.8) | | 32 (16.2) | | 6 (3.0) | *0.033* |
|  | 1 | 60 (73.2) | | 20 (24.4) | | 2 (2.4) |  |
|  | 2 | 46 (68.7) | | 17 (25.4) | | 4 (6.0) |  |
| M |  |  |  |  | |  |  |
|  | 0 | 229 (78.4) | | 53 (18.2) | | 10 (3.4) | 0.134 |
|  | 1 | 37 (67.3) | | 16 (29.1) | | 2 (3.6) |  |
| TNM-stage |  |  |  |  | |  |  |
|  | I | 45 (88.2) | | 4 (7.8) | | 2 (3.9) | *0.011* |
|  | II | 111 (79.3) | | 25 (17.9) | | 4 (2.9) |  |
|  | III | 72 (74.2) | | 21 (21.6) | | 4 (4.1) |  |
|  | IV | 38 (64.4) | | 19 (32.2) | | 2 (3.4) |  |
| Tumor location (right/left) |  |  |  |  | |  |  |
|  | Right | 186 (78.2) | | 45 (18.9) | | 7 (2.9) | 0.284 |
|  | Left | 80 (73.4) | | 24 (22.0) | | 5 (4.6) |  |
| Grading (WHO) |  |  |  |  | |  |  |
|  | low | 208 (75.1) | | 60 (21.7) | | 9 (3.2) | 0.330 |
|  | high | 58 (82.9) | | 9 (12.9) | | 3 (4.3) |  |
| Venous invasion |  |  |  |  | |  |  |
|  | 0 | 211 (77.0) | | 51 (18.6) | | 12 (4.4) | 0.690 |
|  | 1 | 55 (75.3) | | 18 (24.7) | | 0 (0.0) |  |
| Lymphatic invasion |  |  | |  | |  |  |
|  | 0 | 173 (82.0) | | 31 (14.7) | | 7 (3.3) | *0.014* |
|  | 1 | 93 (68.4) | | 38 (27.9) | | 5 (3.7) |  |
| Mucinous (y/n) |  |  | |  | |  |  |
|  | yes | 23 (95.8) | | 1 (4.2) | | 0 (0.0) | *0.026* |
|  | no (NOS) | 243 (75.2) | | 68 (19.9) | | 12 (3.5) |  |
| MMR-status |  |  | |  | |  |  |
|  | proficient | 175 (73.2) | | 55 (23.0) | | 9 (3.8) | *0.040* |
|  | deficient | 62 (84.9) | | 10 (13.7) | | 1 (1.4) |  |
| *KRAS* |  |  |  |  | |  |  |
|  | wildtype | 27 (55.1) | | 18 (36.7) | | 4 (8.2) | *0.042* |
|  | mutated | 34 (77.3) | | 8 (18.2) | | 2 (4.5) |  |

Statistically significant values are indicated in italics.

Abbreviations: TNM -Tumor node metastasis, WHO - World Health Organization, NOS - not otherwise specified, MMR - mismatch repair, *KRAS* – Kirsten rat sarcoma
