# Supplementary material for: Programmed death ligand 1 (PD-L1) in colon cancer and its interaction with budding and tumor-infiltrating lymphocytes (TILs) as tumor-host antagonists
Source: Int J Colorectal Dis. 2021 Jun 25;36(11):2497–510. doi: 10.1007/s00384-021-03985-9 (PMC8505298; doi:10.1007/s00384-021-03985-9)
Supplement: Supplementary file 3 — Supplementary file3 (DOCX 16 KB) [file 384_2021_3985_MOESM3_ESM.docx]

| **Feature** |  | **Low TILs (<5%)**  **(n/%)** | | **High TILs (>5%)**  **(n/%)** | **p-value** |
| --- | --- | --- | --- | --- | --- |
|  |  | 76 | | 74 | 0.853 |
| Age (yrs) | mean |  |  |  |  |
| Gender |  |  |  |  |  |
|  | male | 96 (59.6) | | 65 (40.4) | *0.002* |
|  | female | 81 (43.5) | | 105 (56.5) |  |
| pT |  |  |  |  |  |
|  | 1 | 5 (21.7) | | 18 (78.3) | *<0.001* |
|  | 2 | 20 (51.3) | | 19 (48.7) |  |
|  | 3 | 97 (47.1) | | 109 (52.9) |  |
|  | 4 | 55 (69.6) | | 24 (30.4) |  |
| pN |  |  |  |  |  |
|  | 0 | 85 (42.9) | | 113 (57.1) | *<0.001* |
|  | 1 | 42 (51.2) | | 40 (48.8) |  |
|  | 2 | 50 (74.6) | | 17 (25.4) |  |
| M |  |  |  |  |  |
|  | 0 | 135 (46.2) | | 157 (53.8) | *<0.001* |
|  | 1 | 42 (76.4) | | 13 (23.6) |  |
| TNM-stage |  |  |  |  |  |
|  | I | 21 (41.2) | | 30 (58.8) | *<0.001* |
|  | II | 59 (42.1) | | 81 (57.9) |  |
|  | III | 52 (53.6) | | 45 (46.4) |  |
|  | IV | 45 (76.3) | | 14 (23.7) |  |
| Tumor location (right/left) |  |  |  |  |  |
|  | Right | 121 (50.8) | | 117 (49.2) | 0.927 |
|  | Left | 56 (51.4) | | 53 (48.6) |  |
| Grading (WHO) |  |  |  |  |  |
|  | low | 137 (49.5) | | 140 (50.5) | 0.252 |
|  | high | 40 (57.1) | | 30 (42.9) |  |
| Venous invasion |  |  |  |  |  |
|  | 0 | 127 (46.4) | | 147 (53.6) | *0.001* |
|  | 1 | 50 (68.5) | | 23 (31.5) |  |
| Lymphatic invasion |  |  | |  |  |
|  | 0 | 92 (43.6) | | 119 (56.4) | *0.001* |
|  | 1 | 85 (62.5) | | 51 (37.5) |  |
| Mucinous (y/n) |  |  | |  |  |
|  | yes | 21 (87.5) | | 3 (12.5) | *<0.001* |
|  | no (NOS) | 156 (48.3) | | 167 (51.7) |  |
| MMR-status |  |  | |  |  |
|  | proficient | 124 (51.9) | | 115 (48.1) | 0.702 |
|  | deficient | 36 (49.3) | | 37 (50.7) |  |
| *KRAS* |  |  |  |  |  |
|  | wildtype | 31 (63.3) | | 18 (36.7) | 0.468 |
|  | mutated | 31 (70.5) | | 13 (29.5) |  |

Statistically significant values are indicated in italics.

Abbreviations: TNM - Tumor node metastasis, WHO - World Health Organization, NOS - not otherwise specified, MMR mismatch repair, *KRAS* - Kirsten rat sarcoma
