# Supplementary material for: Programmed death ligand 1 (PD-L1) in colon cancer and its interaction with budding and tumor-infiltrating lymphocytes (TILs) as tumor-host antagonists
Source: Int J Colorectal Dis. 2021 Jun 25;36(11):2497–510. doi: 10.1007/s00384-021-03985-9 (PMC8505298; doi:10.1007/s00384-021-03985-9)
Supplement: Supplementary file 4 — Supplementary file4 (DOCX 15 KB) [file 384_2021_3985_MOESM4_ESM.docx]

| **Feature (n/%)** | **Low buds/high TILs**  **(n=129)** | **Low buds/low TILs**  **(n=128)** | **High buds/high TILs**  **(n=42)** | **High buds/low TILs (n=48)** | **p-value** |
| --- | --- | --- | --- | --- | --- |
| pT stage  1  2  3  4 | 17 (73.9)  14 (35.9)  81 (39.3)  17 (21.5) | 5 (21.7)  18 (46.2)  69 (33.5)  36 (45.6) | 1 (2.4)  5 (12.8)  29 (14.1)  7 (8.9) | 0 (0.0)  2 (5.1)  27 (13.1)  19 (24.1) | *<0.001* |
| pN  0  1  2 | 91 (46.0)  24 (29.3)  14 (20.9) | 67 (33.8)  30 (36.6)  31 (46.3) | 22 (11.1)  17 (20.7)  3 (4.5) | 18 (9.1)  11 (13.4)  19 (28.4) | *<0.001* |
| M  0  1 | 122 (41.8)  7 (12.7) | 100 (34.2)  28 (50.9) | 35 (12.0)  7 (12.7) | 35 (12.0)  13 (23.6) | *<0.001* |
| Mucinous  Yes  No (NOS) | 3 (12.5)  126 (39.0) | 20 (83.3)  108 (33.4) | 0 (0.0)  42 (13.0) | 1 (4.2)  47 (14.6) | *<0.001* |
| Grading (WHO)  Low  High | 102 (36.8)  27 (38.6) | 98 (35.4)  30 (42.9) | 39 (14.1)  3 (4.3) | 38 (13.7)  10 (14.3) | 0.148 |
| TNM-stage  I  II  III  IV | 26 (51.0)  64 (45.7)  31 (32.0)  8 (13.6) | 19 (37.3)  44 (31.4)  37 (38.1)  28 (47.5) | 4 (7.8)  17 (12.1)  14 (14.4)  7 (11.9) | 2 (3.9)  15 (10.7)  15 (15.5)  16 (27.1) | *<0.001* |
| Venous invasion (V)  0  1 | 111 (40.5)  18 (24.7) | 92 (33.6)  36 (49.3) | 37 (13.5)  5 (6.8) | 34 (12.4)  14 (19.2) | *0.008* |
| Lymphatic vessel invasion (L)  0  1 | 95 (45.0)  34 (25.0) | 75 (35.5)  53 (39.0) | 24 (11.4)  18 (13.2) | 17 (8.1)  31 (22.8) | *<0.001* |
| *KRAS*  wildtype  mutated | 8 (16.3)  9 (20.5) | 17 (34.7)  22 (50.0) | 10 (20.4)  5 (11.4) | 14 (28.6)  8 (18.2) | 0.290 |
| MMR status  proficient  deficient | 83 (34.7)  31 (42.5) | 86 (36.0)  30 (41.1) | 33 (13.8)  6 (8.2) | 37 (15.5)  6 (8.2) | *0.041* |

Statistically significant values are indicated in italics.

Abbreviations: NOS – not otherwise specified, WHO – World Health Organization, *KRAS* – Kirsten rat sarkoma, MMR – mismatch repair
